# Effect of Pulsed Electric Fields on the Growth and Acidification Kinetics of *Lactobacillus delbrueckii* Subsp. *bulgaricus*

**DOI:** 10.3390/foods9091146

**Published:** 2020-08-20

**Authors:** Kaidi Peng, Mohamed Koubaa, Olivier Bals, Eugène Vorobiev

**Affiliations:** 1 Sorbonne University, Université de Technologie de Compiègne, ESCOM, EA 4297 TIMR, Centre de Recherche Royallieu, CS 60319, CEDEX 60203 Compiègne, France; kaidi.peng@utc.fr (K.P.); olivier.bals@utc.fr (O.B.); eugene.vorobiev@utc.fr (E.V.); 2 ESCOM, UTC, EA 4297 TIMR, 1 Allée du Réseau Jean-Marie Buckmaster, 60200 Compiègne, France

**Keywords:** fermentation, *Lactobacillusdelbrueckii* subsp. *bulgaricus* CFL1, pulsed electric fields, growth kinetic, acidification activity

## Abstract

The aim of this work was to investigate the effect of pulsed electric fields (PEF) on the growth and acidification kinetics of *Lactobacillus delbrueckii* subsp. *bulgaricus* CFL1 during fermentation. The PEF treatments were applied during the fermentation process using a recirculation pump and a PEF treatment chamber coupled with a PEF generator. The medium flow rate through the chamber was first optimized to obtain the same growth and acidification kinetics than the control fermentation without medium recirculation. Different PEF intensities (60–428 V cm^−1^) were then applied to the culture medium to study the impact of PEF on the cells’ behavior. The growth and acidification kinetics were recorded during the fermentation and the specific growth rates µ, pH, and acidification rate (dpH/dt) were assessed. The results obtained showed a biphasic growth by applying high PEF intensities (beyond 285 V cm^−1^) with the presence of two maximal specific growth rates and a decrease in the acidification activities. It was demonstrated that the cells were stressed during the PEF treatment, but presented an accelerated growth after stopping it, leading thereby to similar absorbance and pH at the end of the fermentation. These results show the great potential of PEF technology to be applied to generate low acidified products by performing PEF-assisted fermentations.

## 1. Introduction

Lactic acid bacteria (LAB) have extensive industrial applications as producers of lactic acid, as probiotics, as biocontrol agents, and as bio preservatives [1]. The main application of LAB is as starter cultures in the food industry with a wide variety of fermented dairy products, meat, fish, fruit, vegetable, and cereal products [2]. LAB have numerous metabolic characteristics such as the acidification activity, proteolytic activity, synthesis of bacteriocin, and production of exopolysaccharides that contribute to the nutritional value and organoleptic quality and extended the shelf life of food products [3,4,5,6]. The strain *Lactobacillus delbrueckii* subsp. *bulgaricus* is a representative lactic acid bacterium that is extensively used for the production of the most popular types of fermented milk [7,8]. It can convert carbohydrates, such as glucose and lactose, to lactic acid [9], which may contribute to the texture and aroma of different food products [10].

The volume of the fermenters for the production of LAB at industrial scale varies according to the installations, from a few hundred liters to a few tens of thousands of liters (10 to 30 m^3^) [11]. The fermentation is carried out under controlled conditions which, when known, correspond to the optimal conditions for each bacterial species, and most often to the know-how of the producer. However, for a given species, these conditions differ according to the strain considered, the property of interest (e.g., growth rate, growth yield, production of lactic acid, etc.), and the method of determining this property [12]. Regardless of the composition of the culture medium, the main experimental conditions to be taken into account are the stirring speed, the temperature, the pH and the type of neutralizer used for regulating the pH, the atmosphere and/or the redox potential, and the water activity. These environmental conditions also have an effect on the survival and resumption of cell activity after harvesting and stabilization [11].

Besides changing the medium composition and the fermentation parameters to affect the growth and product kinetics, some research works have been interested in changing the culture conditions by applying non-conventional technologies that induce stress to microorganisms before or during the fermentation. Among these technologies, pulsed electric fields (PEF), ultrasounds (US), and high-hydrostatic pressure (HHP), are the most investigated [13]. These technologies are usually used in food processing either to inactivate the contaminating microorganisms [14] or to intensify the extraction of valuable molecules from the intracellular compartments [15,16]. However, when applied at sub-lethal levels, they may change the fermentation kinetics by, for example, reducing the fermentation time and producing the molecule of interest differently, compared to the non-stressed cultures [17].

One of the promising technologies to stress microorganisms is PEF. This technology has the ability to induce the formation of pores in the cell membranes and cause the breakdown of the cells, which can be reversible or irreversible, depending mainly on the applied PEF parameters, the medium composition, and the cell type [18,19]. PEF treatments usually involve the application of high intensity pulses (typically > 20 kV cm^−1^) for a treatment time less than 1 s [20]. These high intensities are generally used for microbial inactivation in food products. Other interesting applications of PEF, when applied at sub-lethal levels, have been investigated for different types of microorganisms, including LAB [21,22,23]. For example, it was reported that the application of PEF to *Lactococcus lactis* subsp. *cremoris* strains at a field intensity of 8 kV cm^−1^, a pulse length of 1 µs, and a number of pulses of 200, increased the exopolysaccharide (EPS) yield by 32% in a culture medium containing 1% lactose. In another work, PEF treatment of *Lactobacilli* cells at a field intensity of 7.5 kV cm^−1^ for 3.5 ms showed an enhanced β-glucosidase activity and an increased bioconversion of isoflavone glucosides to aglycones, compared to the untreated culture [24]. Furthermore, the mild PEF-treated strains *Lactobacillus delbrueckii* subsp. *bulgaricus* LB−12 and *Lactobacillus acidophilus* LA-K demonstrated higher acid tolerance, compared to the untreated ones [8]. Likewise, the surviving population of *Lactobacillus plantarum* 564 after PEF treatment was able to grow in de Man, Rogosa and Sharpe (MRS) broth and showed an increased resistance to further PEF treatment [25].

Despite of the interesting results of LAB stimulation by PEF showing increased products’ formation and tolerance, investigating the acidification and growth kinetics during fermentation was not performed before. In addition, little information is available in the literature regarding the mechanisms of PEF microbial stimulation. Hence, the aim of this work was to evaluate the impact of PEF treatment on the growth and acidification activity of *L*. *delbrueckii* subsp. *bulgaricus* CFL1 in MRS broth during fermentation.

## 2. Materials and Methods

### 2.1. Bacterial Strain and Preculture Conditions

The strain used in this work is *Lactobacillus delbrueckii* subsp. *bulgaricus* CFL1. It was kindly provided by the laboratory “Génie et Microbiologie des Procédés Alimentaires (GMPA)” (INRAE, Thiverval-Grignon, France). Stock cells of 1 mL aliquots were stored in 2-mL cryotubes at −80 °C in de Man, Rogosa and Sharpe (MRS) broth (VWR International SAS, Fontenay-sous-Bois, France) supplemented with 15% glycerol. The precultures were prepared by adding aseptically, in a 100-mL glass bottle, 1 mL of the stock cells into 30 mL of sterile MRS broth, and incubating at 42 °C without agitation until reaching an optical density at 600 nm (OD_600nm_) of 3.3 ± 0.2.

### 2.2. Fermentation Conditions

#### 2.2.1. Control Fermentation

**Figure 1:**
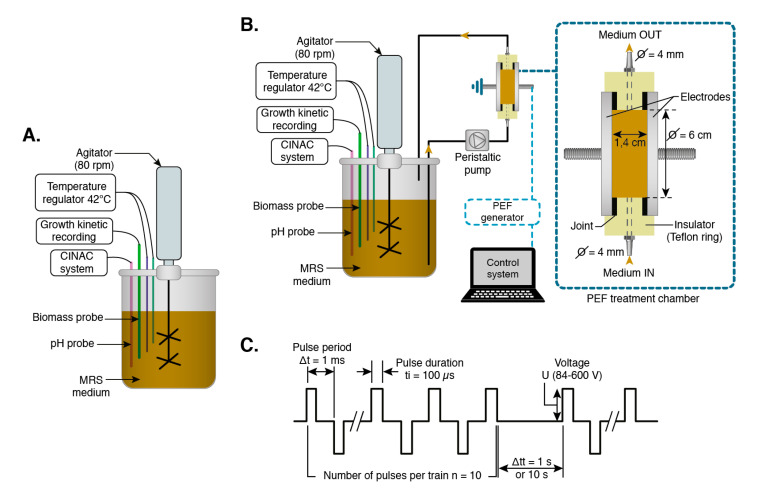
(**A**) Control fermentation. (**B**) pulsed electric fields (PEF)-assisted fermentation of *L. delbrueckii* subsp. *bulgaricus* CFL1. CINAC, acidification activity measurement. (**C**) PEF treatment parameters applied during the fermentation of *L. delbrueckii* subsp. *bulgaricus* CFL1.

A volume of 1.5 L MRS broth (VWR International SAS, Fontenay-Sous-Bois, France) was prepared as growth medium according to the manufacturer instructions, and then introduced into a 2-L anaerobic bioreactor. After sterilization at 121 °C for 20 min and cooling to ambient temperature, the agitation and temperature were set at 80 rpm and 42 °C, respectively, whereas the initial pH of the medium was adjusted to 6.3 with NaOH (1 M). The medium was then inoculated using the preculture prepared to get an initial OD_600nm_ of 0.03 in the bioreactor. During the 24 h of fermentation, the growth kinetics were recorded every minute using a biomass probe (EXcell 231 NIR biomass sensor Ø12mm, CellD, Roquemaure, France) monitored by Expert system software (CellD, Roquemaure, France), whereas the pH was recorded using a CINAC system [26] kindly provided by the laboratory “Génie et Microbiologie des Procédés Alimentaires (GMPA)” (INRAE, Thiverval-Grignon, France) (Figure 1A). The pH was recorded every minute and the acidification rate (dpH/dt, in min^−1^) was calculated with the WCIDUS software (INRAE, Thiverval-Grignon, France). Before introduction into the bioreactor, the biomass and pH probes were disinfected by ethanol (75%) and rinsed with sterile water. The probes were placed in the medium under aseptic conditions and connected tightly to the bioreactor to ensure the anaerobic environment during the fermentation. The specific growth rate µ (min^−1^) was determined by calculating the first derivative of the equation of the Ln (A) variation versus time [27,28] using Origin 85 software.

#### 2.2.2. PEF-Assisted Fermentation

PEF-assisted fermentation was conducted using a continuous flow treatment chamber consisting of two round-edged disk-shaped stainless steel electrodes with parallel assembly and separated by an insulating Teflon ring having two holes that allow the medium circulation through the chamber (Figure 1B). The distance between the electrodes was set to 1.4 cm, providing a volume inside the PEF-treatment chamber of 40 mL (a ratio of 1:50 between the volume of the bioreactor and that of the treatment chamber). The medium was re-circulated continuously from the bioreactor to the PEF-treatment chamber and back into the bioreactor using a peristaltic pump that controls the medium flow rate. The bioreactor was connected to the treatment chamber by 4-mm silicone tubes and the assembly was sterilized as above-mentioned before fermentation. The circulation flow rate was first optimized (two circulation flow rates of 25 mL min^−1^ and 50 mL min^−1^ were tested) to allow obtaining the same growth and acidification kinetics compared to the control fermentation (Figure 1A).

The PEF treatments were then applied during the fermentation of *L. delbrueckii* subsp. *bulgaricus* CFL1. The two electrodes were connected to a PEF generator of 5 kV and 1 kA (Hazemeyer, Saint-Quentin, France), providing monopolar pulses of near-rectangular shape [29]. The PEF voltages applied were of 84, 126, 400, and 600 V. Taking into account the distance between the electrodes of 1.4 cm, the respective intensities were of 60, 90, 285, and 428 V cm^−1^. The number of pulses was fixed to *n* = 10 by train, the pulse period was Δ*t* = 1 ms, the pulse duration was *t_i_* = 100 µs, and the time between the trains was fixed to either Δ*t_t_* = 1 s or Δ*t_t_* = 10 s. The PEF treatments were carried out from the beginning of the fermentation and lasted for either 120 min or 240 min. The fermentation was then continued up to 1440 min (24 h) without PEF treatment (Figure 1C). The effective PEF treatment time *t_PEF_* was calculated as follow: *t_PEF_* (s) = *N·n·t_i_* (s) [17], where *N* is the number of trains, *n* is the number of pulses per train, and *t_i_* is the pulse duration.

### 2.3. Statistical Analysis

The average values and standard deviations in the graphs were calculated using three biological replicates. One-way analysis of variance (ANOVA) was used to determine the significant differences using StatPlus V6 software for Macintosh systems.

## 3. Results and Discussion

### 3.1. Effect of Recirculation Flow Rate on the Growth and Acidification Kinetics

**Figure 2:**
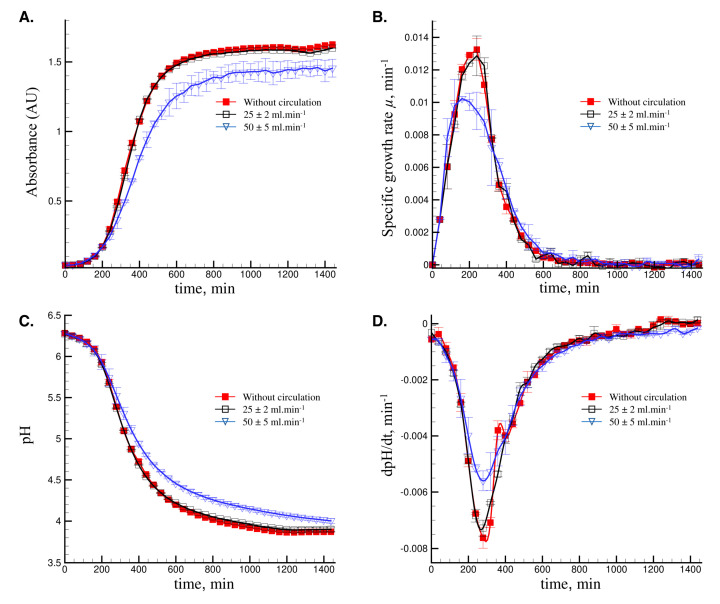
Growth and acidification characteristics of *L. delbrueckii* subsp. *bulgaricus* CFL1 in de Man, Rogosa and Sharpe (MRS) broth with different circulation flow rates. The fermentations were carried out with the initial OD_600nm_ of 0.03. (**A**) The absorbance (AU) of the media, (**B**) the specific growth rates µ of the media, (**C**) the pH values of the media, (**D**) the acidification rates (dpH/dt) of the media. The error bars represent the standard deviations of the three biological replicates.

The impact of medium circulation through the treatment chamber without PEF application was studied on the growth and acidification kinetics of *L. delbrueckii* subsp. *bulgaricus* CFL1 at the flow rates of 25 ± 2 mL min^−1^ and 50 ± 5 mL min^−1^. The results were compared to those obtained without medium circulation and are presented in Figure 2.

The results in Figure 2A show that a medium circulation of 25 ± 2 mL min^−1^ allows obtaining a similar growth kinetic compared to the control without medium circulation, whereas both a lower growth rate and final absorbance value were observed at the flow rate of 50 ± 5 mL min^−1^. The comparison between the graphs of the specific growth rates (Figure 2B) shows similar curves between the control cells and those circulated at 25 ± 2 mL min^−1^, whereas a different curve was obtained for the cells circulated at 50 ± 5 mL min^−1^. The maximum specific growth rate µ_max_ values were, therefore, 0.0132 ± 0.0068 min^−1^ and 0.0129 ± 0.0012 min^−1^ for the control cells and those circulated at 25 ± 2 mL min^−1^, respectively. These values were significantly higher (*p* < 0.05) than those of the cells circulated at 50 ± 5 mL min^−1^, of 0.0095 ± 0.0011 min^−1^.

The results of the growth kinetics were supported by those of the acidification kinetics. The graphs in Figure 2C,D show a lower acidification rate when a circulation flow rate of 50 ± 5 mL min^−1^ was applied, compared to the control culture and that applying a flow rate of 25 ± 2 mL min^−1^. The variations in the specific growth rate (Figure 2B) and acidification rate (Figure 2D) followed a similar pattern consisting of increasing to a maximum value and steadily decreasing with the progress of fermentation. This result concurs with that of Mercier et al. [30] who reported that the volumetric rates of biomass production and lactic acid biosynthesis are closely associated and followed similar patterns during glucose fermentation by *Latobacillus amylophilus*.

The impact of medium agitation on LAB growth and product synthesis has been already shown in the literature. For instance, *Lactococcus lactis* subsp. *cremoris* strain showed a delay in the growth and metabolite exopolysaccharide (EPS) production when applying agitation or circulation of the medium during fermentation [21]. A decrease of 22.5% in the growth rate of the strain *Lactobacillus plantarum* was observed during the fermentation of edible Irish brown seaweeds by increasing the agitation speed from 0 to 100 rpm [31]. Increasing the agitation rate induces different aeration conditions in the medium, which impact the fermentation performances. *L. delbrueckii* subsp. *bulgaricus,* used in this work, is an aerotolerant anaerobe that does not require strict anaerobic growth conditions or use oxygen in its energy metabolism [32]. It is likely that the presence of oxygen in its environment can influence its physiology [33]. The differences in the growth rates might be attributed to the differences in the metabolic pathways under aerobic and anerobic conditions [34]. Oxygen inhibition associated with superoxide was supposed to be another reason for the lower growth rate [35]. At a circulation flow rate of 50 ± 5 mL min^−1^, significantly (*p* < 0.05) lower final cell biomass absorbance (1.42) was observed compared to the control (1.64) and that applying a flow rate of 25 ± 2 mL min^−1^ (1.63). It was previously reported that increasing the micro-aeration level from 0 to 0.10 vvm increased the cell number; however, its further increase up to 0.15 vvm decreased the maximum cell number [36]. Besides the stress induced to the cells during the circulation of the medium through the treatment chamber, the increase in the dissolved oxygen concentration could be also responsible for the lower growth and acidification rates when circulating the medium at 50 ± 5 mL min^−1^. This observation concurs with that reported by Jeanson et al. [37].

### 3.2. The Impact of PEF Treatment on the Growth and Acidification Kinetics

#### 3.2.1. The Impact of PEF Intensities

**Figure 3:**
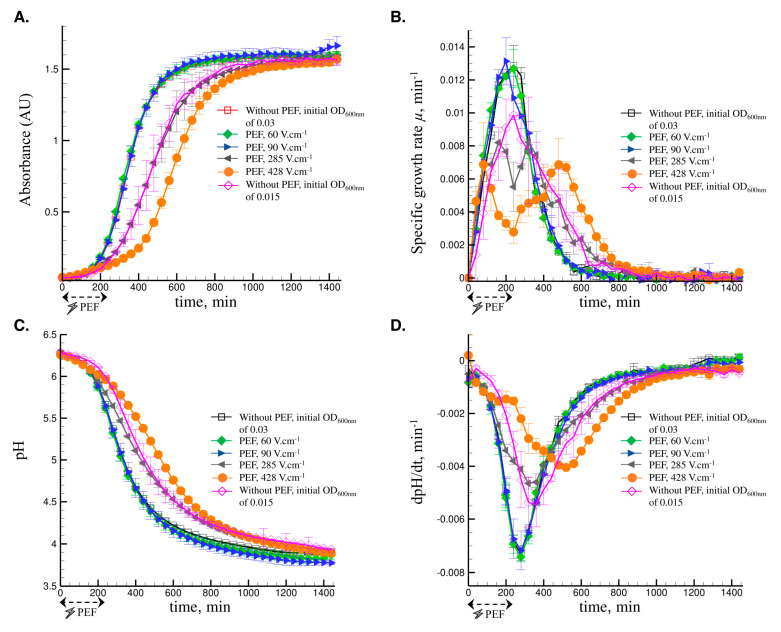
Growth and acidification kinetics of *L. delbrueckii* subsp. *bulgaricus* CFL1 in MRS broth with a medium circulation flow rate of 25 ± 2 mL min^−1^. □ ♦ ► ◄ ●, fermentations performed with the initial OD_600nm_ of 0.03 without PEF treatment, with PEF treatment at voltages of 60, 90, 285, 428 V cm^−1^, respectively. ◊, fermentation performed with less inoculum giving an initial OD_600nm_ of 0.015 without PEF treatment. (**A**) Absorbance (AU) of medium determined using the biomass probe, (**B**) specific growth rate µ, (**C**) pH values of medium, (**D**) acidification rate dpH/dt. Vertical error bars represent the standard deviation of three biological replicates.

The PEF-assisted fermentations were performed using the medium circulation flow rate of 25 mL min^−1^, which allows investigating the impact of PEF treatment on the growth and acidification kinetics. The results in Figure 3 show no impact of PEF treatment on the growth and acidification kinetics of *L. delbrueckii* subsp. *bulgaricus* CFL1 when applying low PEF intensities (60, 90 V cm^−1^). However, increasing the intensities to 285 V cm^−1^ and 428 V cm^−1^ led to a biphasic growth of the cells, while no significant changes in the final biomass concentrations were observed, regardless of the intensity applied. Interestingly, the specific growth rate curves (Figure 3B) showed two peaks at the same level for either the PEF-assisted fermentations at 285 V cm^−1^ or 428 V cm^−1^, with lower values for the PEF-assisted fermentation at 428 V cm^−1^ compared to those at 285 V cm^−1^. The first peak appears during the 240 min of the PEF treatment, whereas the second one appears after stopping the PEF treatment (beyond 240 min). It seems like it obtained two growth curves, the first one during the PEF treatment, showing a high stress of the cells with limited cell growth, and a second one after stopping the PEF treatment, showing the recovery of the normal cell growth. The negative impact of PEF treatment on the cell growth was linked to its intensity for 285 V cm^−1^ and 428 V cm^−1^. It should be noted that despite of the stress induced by the PEF treatments to the cells, similar final biomass concentrations were obtained for the control and all the PEF-assisted fermentations. Similar results were obtained by Seratlić et al. [25], who reported that the growth of *L. plantarum* 564 was resumed after PEF treatment, with a higher growth rate observed during the late-growth phase, compared to the untreated cells.

To understand the behavior obtained for *L. delbrueckii* subsp. *bulgaricus* CFL1 during PEF-assisted fermentation, a control culture with medium circulation and without PEF treatment was inoculated to get an initial OD_600nm_ in the bioreactor of 0.015 instead of 0.03. The results showed delayed growth and acidification kinetics compared to the experiments starting with an OD_600nm_ of 0.03. This is due to the smaller amount of biomass inoculated in the bioreactor. Results in Figure 3B show the presence of only one peak of specific growth rate, similar to the other experiments without PEF treatment. This confirms the biphasic growth of the cells when applying PEF treatment, which exerts an inhibitory effect of the growth and possible sublethal injuries during the PEF treatment, but at the same time stimulating the cells for faster growth after stopping the PEF treatment. The same correlation was observed for the acidification activity showing the presence of only one peak of the acidification rate when starting the fermentation with an OD_600nm_ of 0.015 (Figure 3D).

Regarding the acidification activities, results in Figure 3C,D show lower rates and delayed activities when applying PEF treatment at 285 V cm^−1^ and 428 V cm^−1^, compared to either the control or the treated cells at 60 V cm^−1^ and 90 V cm^−1^. Similar to the growth kinetics, the PEF treatments at 285 V cm^−1^ and 428 V cm^−1^ impacted the acidification kinetics. In fact, during the 240 min of PEF treatment, the cells were stressed with a very low acidification kinetic recorded (for both higher PEF intensities), followed by a fast recovery of the acidification activity after stopping the PEF treatment. This result shows again the stress induced by PEF treatment on the cells during its application and the fast recovery of the activities after stopping it. It should be also noted, similarly to the growth kinetics, that after 24 h fermentation, PEF treatment applied to the culture for the first 240 min did not show any impact on the final pH value, which could be explained by the faster growth observed beyond this point. These results concur with the ones reported in the literature. For instance, Ewe et al. [38] showed that the PEF treatment delayed the cell growth but maintained or enhanced the metabolic activity of lactic acid bacteria. Results on the impact of PEF on the growth and acidification rates are summarized on Table 1.

#### 3.2.2. Effect of the Treatment Time and the Duration between the Trains

**Figure 4:**
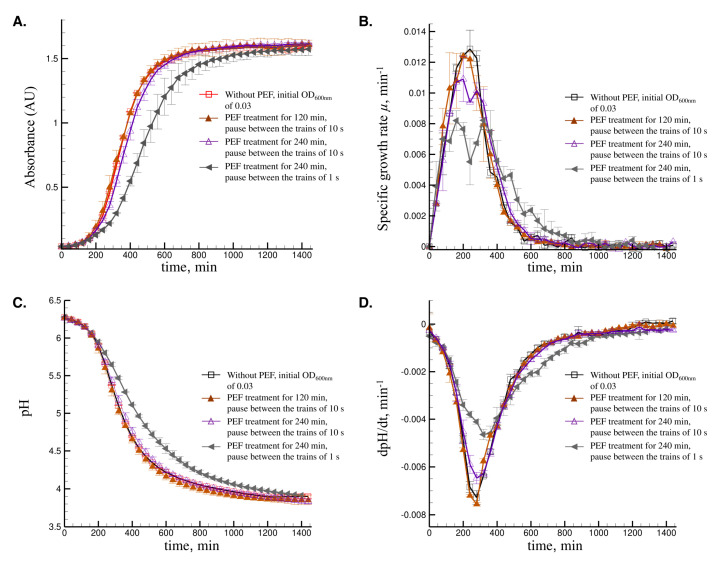
Growth and acidification kinetics of *L. delbrueckii* subsp. *bulgaricus* CFL1 in MRS broth under different PEF treatment parameters. Fermentations were performed with an initial OD_600nm_ of 0.03. □, kinetics without PEF treatment. PEF-assisted fermentations were performed at the intensity of 285 V cm^−1^. ▲, PEF treatment for 120 min with a pause between the trains of 10 s. ∆, PEF treatment for 240 min with a pause between trains of 10 s. ◄, PEF treatment for 240 h with a pause between trains of 1 s. (**A**) Absorbance (AU) of the medium, (**B**) Specific growth rate *µ*, (**C**) pH values of the medium, (**D**) Acidification rate dpH/dt. Vertical error bars represent the standard deviation of three biological replicates.

The impact of PEF treatment time and the duration between the trains was also studied during the fermentation of *L. delbrueckii* subsp. *bulgaricus* CFL1. A pause between the trains of 10 s was applied for the PEF treatment during 120 min, whereas both 10 s and 1 s pauses were applied for the PEF treatment during 240 min. The effective PEF treatment time, *t_PEF_*, was of 0.72, 1.44, and 14.28 s, respectively. Results in Figure 4 show that the impact of PEF treatment on the growth and acidification kinetics was more pronounced by increasing the treatment time from 120 to 240 min, and by decreasing the pause between the trains from 10 to 1 s. Treating the cells with PEF for 120 min did not significantly affect the growth and acidification kinetics, compared to the control, without PEF treatment. Increasing the PEF treatment time up to 240 min, with 10 s between the trains, slightly impacted the growth and the acidification kinetics. A biphasic growth started to appear, which was clearly shown by the presence of 2 peaks in the curves of the specific growth rate (Figure 4B), and a reduced acidification rate (Figure 4C). These peaks were more pronounced when decreasing the pause between the trains from 10 to 1 s, which means that the cells were more stressed by PEF.

## 4. Conclusions

In this work, the impact of PEF during the fermentation of *Lactobacillus delbrueckii* subsp. *bulgaricus* was investigated. It could be concluded from the results obtained that PEF treatment stressed the cells, which was shown by a biphasic growth and a reduced acidification activity. The impact of PEF treatment could be beneficial in the case of a lower acidification activity is sought by these LAB, which may open the way towards novel applications in different industrial fields. The mechanisms behind obtaining a biphasic growth and a delayed acidification activity remain, nevertheless, not fully understood and could be associated with sublethal injuries of the cells and to metabolic changes related to PEF applications. Despite the interesting results obtained, further research works are needed to investigate the impact of other PEF parameters on LAB, such as the pulse duration and frequency, and to study the effect of PEF treatment on the cell integrity during PEF-assisted fermentation, which contributes to a better understanding of the impact of PEF applied at sub-lethal levels.

## Figures and Tables

**Table 1:** Impact of PEF treatment on the growth and acidification rates.

		µ_max_ (min^−1^)		dpH/dt (min^−1^)	
Initial OD_600nm_	Treatment Intensity (V cm^−1^)	Average	SD	Average	SD
0.015	0	0.0099	0.0001	−0.0055	0.0008
0.03	0	0.0129	0.0012	−0.0073	0.0003
0.03	60	0.0127	0.0011	−0.0074	0.0002
0.03	90	0.0131	0.0014	−0.0072	0.0007
0.03	285	0.0082; 160 min	0.0007	−0.0046	0.0002
		0.0082; 320 min	0.0006		
0.03	428	0.0069; 80 min	0.0025	−0.0040	0.0000
		0.0069; 480 min	0.0016		
